# Aging and brain connectivity by graph theory

**DOI:** 10.18632/aging.203680

**Published:** 2021-11-02

**Authors:** Fabrizio Vecchio

**Affiliations:** 1 Brain Connectivity Laboratory, Department of Neuroscience and Neurorehabilitation, IRCCS San Raffaele Roma, Rome, Italy; 2 eCampus University, Novedrate (Co), Rome, Italy

**Keywords:** aging, EEG, graph theory, small world, connectome

Physiological and pathological brain aging plays a central role in brain networks modulation. Aging is a multifactorial physiological process characterized by the accumulation of degenerative processes impacting on different brain functions, including the cognitive one. A tool largely employed in the investigation of brain networks is the electroencephalogram (EEG).

Moving from the hypothesis that aging processes modulate brain connectivity networks, modern analysis of EEG rhythms provides information on dynamic brain connectivity. Recently, graph theory functions were applied in several studies on EEG cortical sources in order to evaluate specific parameters such as small-world as a representative model of network architecture.

Network science and graph theory applications have recently spread widely to help in understanding how human aging condition are linked to neuronal network structure, thus providing a conceptual frame that can help in reducing the analytical brain complexity and underlining how network topology can be used to characterize and model processes of aging, brain disease and dysfunction [1].

This editorial summarizes methodological advances in this field. Electroencephalographic functional network studies in physiological aging will be presented to discuss whether network science is changing the traditional concept of brain aging and how network topology knowledge could help in modeling resilience and vulnerability of ageing and correlated diseases.

In the following mentioned studies, graph theory functions were applied to the undirected and weighted networks obtained by the lagged linear coherence evaluated by exact Low Resolution Electromagnetic Tomography (eLORETA). The following frequency bands: delta (2-4Hz), theta (4-8Hz), alpha1 (8-10.5Hz), alpha2 (10.5-13Hz), beta1 (13-20Hz), beta2 (20-30Hz) and gamma (30-40Hz) were explored in the whole brain or specific networks [2–4].

First of all, to test the hypothesis that aging processes modulate brain connectivity network, EEG was recording on 113 healthy volunteers, divided into three groups according with their ages: 36 Young (15-45years), 46 Adult (50-70years), 31 Elderly (>70years). Normalized Characteristic Path Length (λ) presented the pattern Young>Adult>Elderly in alpha2. Elderly also showed an increase in delta and theta bands. The correlation between age and λ showed that higher ages corresponded to higher λ in delta and theta and lower in the alpha2 band; this pattern reflects the age-related modulation of higher (alpha) and decreased (delta) connectivity [5].

In order to define age-related normative limits, EEG of 170 healthy elderly volunteers were recorded. The small-world parameter was carried out in the whole brain, in the left and the right hemispheres separately, and in three specific resting state subnetworks: attentional network (AN), frontal network (FN), and default mode network (DMN). To evaluate the stability of the investigated parameters, a subgroup of 32 subjects underwent three separate EEG recording sessions in identical environmental conditions after a few days interval. Results showed that the whole and right/left hemispheric evaluation did not present side differences, but when individual subnetworks were considered, AN and DMN presented in general higher SW in low (delta and/or theta) and high (gamma) frequency bands in the left hemisphere, while for FN, the alpha1 band was lower in the left with respect to the right hemisphere. It was also evident the test-retest reliability and reproducibility of the present methodology when carried out in clinically stable subjects [6].

Furthermore, in order to evaluate whether this proposed method for the evaluation of Small World reproducibility and stability, a specific study [7] evaluated 80 subjects: 36 healthy young, 32 healthy elderly, and 12 Alzheimer's patients. EEG were recorded during six separate sessions (480 recordings) at an average inter-session interval of 3.8±0.2days. The results confirmed the good reproducibility and stability and the pattern Young>Elderly>AD in low frequency delta and theta bands and vice versa in the higher alpha band. Moreover, the correlation with age confirmed in healthy subjects that older the age higher the SW values for alpha2.

Finally, given the cerebral complexity and dynamism, many non-linear approaches have also been applied to explore age-related brain network modulation detected by the EEG: one is the entropy, a measure of system disorder. A recent study [8] was aimed to investigate aging influence on brain dynamics applying Approximate Entropy (ApEn) parameter to EEG data of 68 healthy adults, divided with respect to their age in two groups. Results showed that elderly participants present higher ApEn values than younger participants in central, parietal and occipital areas, confirming the hypothesis that aging is characterized by an evolution of brain dynamics. Such findings may reflect a reduced synchronization of the neural networks cyclic activity, due to the reduction of cerebral connections typically found in aging process.

In conclusion, graph theory represents a reliable method to address brain connectivity patterns from EEG data and is particularly suitable to study the physiological impact of aging on brain functional connectivity networks. Evidences from the mentioned studies confirm the stability of the Small World index [5–7] and suggests that graph theory can help in the analysis of connectivity patterns estimated from EEG and can facilitate the study of the physiological and pathological brain aging features of functional connectivity networks. The proposed method for the evaluation of the characteristics of the Small World (SW) has shown good reproducibility and stability and applied to patient data, this technique and the understanding of the dynamics of brain networks by applying the entropy parameter could provide more information on the pathophysiological processes underlying the age-related brain disconnection, as well as on the administration of rehabilitation treatments at the right time that could allow to avoid unnecessary interventions.
